# Apixaban-Induced Spontaneous Hemorrhagic Cardiac Tamponade

**DOI:** 10.7759/cureus.55476

**Published:** 2024-03-04

**Authors:** Jeewan Ambat, Siddharth Patel, Prutha R Pathak, McAnto Antony, Ashish K Basu, Francis G Sto. Domingo, Paul Q Vu

**Affiliations:** 1 Internal Medicine, Alabama College of Osteopathic Medicine, Dothan, USA; 2 Internal Medicine, Decatur Morgan Hospital, Decatur, USA; 3 Internal Medicine, North Alabama Medical Center, Florence, USA; 4 Endocrinology, Diabetes and Metabolism, Medical University of South Carolina, Anderson, USA; 5 Heart Center, Decatur Morgan Hospital, Decatur, USA

**Keywords:** direct oral anticoagulants (doacs), factor xa inhibitor, acute pericardial effusion, spontaneous pericardial effusion, hemorrhagic cardiac tamponade

## Abstract

Direct oral anticoagulants (DOACs), such as apixaban, are used for the prevention and management of thromboembolic diseases. Here, we present a case of a 72-year-old African American woman who presented to the hospital with shortness of breath and precordial chest pain for three days. The patient was diagnosed with volume overload associated with the progression of chronic kidney disease (CKD) and subsequently admitted to the hospital. Since the patient failed to adequately respond to diuretics, hemodialysis was initiated. During the hospital stay, she developed paroxysmal atrial fibrillation. Along with amiodarone, apixaban was started for primary stroke prophylaxis. Within 72 hours, the patient developed worsening chest pain. An echocardiogram revealed a large pericardial effusion with cardiac tamponade. She was taken for an emergent open pericardial window placement to relieve cardiac tamponade, where 600 mL of blood was drained. Considering the timeline of the development of a large bloody pericardial effusion following initiation of apixaban, spontaneous hemorrhagic cardiac tamponade attributed to the use of apixaban was diagnosed. The patient was eventually taken off all anticoagulants. In considering potential mechanisms, impaired hepatic and renal metabolism of apixaban could be factored in this case. In addition, CKD can increase bleeding risk, due to platelet dysfunction and impaired interaction of von Willebrand factor with GPIIb-IIIa. Moreover, renal secretion of apixaban is mediated by p-glycoprotein and amiodarone is an inhibitor of this protein. Although extremely rare, spontaneous hemorrhagic cardiac tamponade can occur with the use of DOACs, such as apixaban. Prompt recognition and urgent treatment remain keys to avoiding adverse patient outcomes.

## Introduction

Rivaroxaban, apixaban, and edoxaban are direct oral anticoagulants (DOACs) used for the prevention and management of thromboembolic diseases (e.g., deep venous thrombosis, pulmonary embolism, stroke prevention in atrial fibrillation, and peripheral arterial disease). The advantages of DOAC therapy compared to other anticoagulation therapies (e.g., heparin and warfarin) include fewer drug-drug interactions and not requiring prothrombin time/international normalized ratio (PT/INR) monitoring. DOACs, however, are known to increase bleeding risk in patients with several comorbidities [1].

Rhee et al. found that among 19 studies comparing the outcomes of DOACs and warfarin in patients with non-valvular atrial fibrillation and renal impairment, apixaban was associated with the lowest risk of major bleeding in patients with advanced chronic kidney disease (CKD) [2]. When DOACs cause bleeding in patients with these comorbidities, the bleeding sites are most commonly gastrointestinal and intracranial [1]. Hemopericardium or hemorrhagic cardiac tamponade due to DOACs is extremely rare [3]. Here, we present a case of spontaneous hemorrhagic cardiac tamponade after the use of apixaban.

## Case presentation

A 72-year-old African American woman presented to the hospital with shortness of breath and precordial chest pain for three days. She reported a constant, non-radiating pain, 3/10 in intensity, without alleviating or exacerbating factors. She reported associated shortness of breath at rest and palpitations. The chest pain resolved when she received aspirin and nitroglycerin en route to the emergency room. Her medical history was significant for stage 4 CKD, stable angina, chronic obstructive pulmonary disease (COPD), anemia, abdominal aortic aneurysm repair with complications leading to paraplegia, and gastric arteriovenous malformation needing cauterization. She had been a one-pack-per-day smoker for 60 years, and she denied alcohol and recreational substance use. She lived at home with her family and was dependent on all activities of daily living.

In the emergency room, her temperature was 98.2°F, blood pressure 148/72 mm Hg, pulse 83 per minute, respiratory rate 22 per minute, and oxygen saturation 98% on two liters nasal cannula. Her physical exam was significant for inspiratory crackles throughout bilateral lower lung fields without wheezing. S1 and S2 were normal without any murmur, rub, or gallop. She had bilateral lower extremity pitting edema that extended to the mid-shins. She was drowsy, requiring frequent verbal and physical stimulation to stay engaged in the clinical encounter. Lab investigations detected elevated pro-BNP and elevated but flat-trend troponin, close to baseline chronic anemia, and worsening uremia (Table 1). Electrocardiogram (EKG) demonstrated a normal sinus rhythm without concerning ST segment or T wave changes. Chest imaging revealed pulmonary edema.

**Table 1:** Electrolyte panel, cardiac enzymes, BMP, and CBC BMP: basic metabolic panel, CBC: complete blood count, BUN: blood urea nitrogen, GFR: glomerular filtration rate, AST: aspartate aminotransferase, ALT: alanine aminotransferase, WBC: white blood cell, RBC: red blood cell, Hgb: hemoglobin, Hct: hematocrit, MCV: mean corpuscular volume, MCH: mean corpuscular hemoglobin, MCHC: mean corpuscular hemoglobin concentration, PT: prothrombin time, INR: international normalized ratio, PTT: partial thromboplastin time, m^2^ = meters raised to the power of two

	4/10/2021	Reference value
Sodium	139	136-145 mmol/L
Potassium	5.2	3.5-5.1 mmol/L
Chloride	106	98-107 mmol/L
Carbon dioxide	18	25-35 mmol/L
Anion gap	15	8-12 mEq/L
BUN	58	8-22 mg/dL
Creatinine	5.7	0.5-0.9 mg/dL
Estimated GFR/1.73 m^2	9	≥90
BUN/creatinine ratio	10	10-20
Glucose	116	70-104 mg/dL
Calculated osmolality	295	275-295 mOsm/kg
Calcium	8.4	8.8-10.2 mg/dL
Total bilirubin	0.30	0.20-1.00 mg/dL
AST	15	10-30 U/L
ALT	11	10-36 U/L
Alkaline phosphatase	95	32-104 U/L
Creatinine kinase	134	24-173 U/L
Troponin T	886	0-19 ng/L
Pro-B-natriuretic peptide	20030	5-353 pg/mL
Total protein	7.8	6.3-8.3 g/dL
Albumin	3.7	3.5-5.0 g/dL
Globulin	4.1	2.0-3.5 g/dL
Albumin/globulin ratio	0.9	1-2
WBC	8.21	4.8-10.8 x 1000
RBC	2.81	4.2-5.4 x MIL
Hgb	7.4	12.0-16.0 g/dL
Hct	24.7	37.0-47.0%
MCV	87.9	81-99 FL
MCH	26.3	27-31 PG
MCHC	30.0	33-37 g/dL
RDW standard deviation	14.9	11.5-14.5%
Platelet count	355	130-400 x 1000
PT	15.4	11.0-16.0
INR	1.20	≤1.1
PTT	33.2	22.3-41.8

The patient was diagnosed with volume overload associated with the progression of CKD and subsequently admitted to the hospital for further management. Cardiology and nephrology were consulted for the management of volume overload, and intravenous (IV) furosemide 200 mg every eight hours was initiated. EKG detected an ejection fraction greater than 60% without wall motion abnormality, trace pericardial effusion without tamponade, and pulmonary hypertension of 50 mmHg. Unfortunately, she failed to respond adequately to diuretics, and ultimately on day four of hospitalization, a fluoroscopy-guided internal jugular hemodialysis catheter was placed. The patient tolerated the procedure without any complications except for a brief episode of atrial fibrillation with rapid ventricular response, which, following an IV bolus of amiodarone 150 mg, converted to a normal sinus rhythm. Post procedure, oral amiodarone 400 mg twice daily was administered, and 24 hours after the procedure, oral apixaban 2.5 mg twice daily was added for primary stroke prophylaxis. The patient tolerated two hemodialysis sessions on the fifth and sixth days of the hospital course, respectively. Subsequently, her clinical status improved with a reduction in drowsiness, shortness of breath, and lower extremity edema.

**Figure 1:**
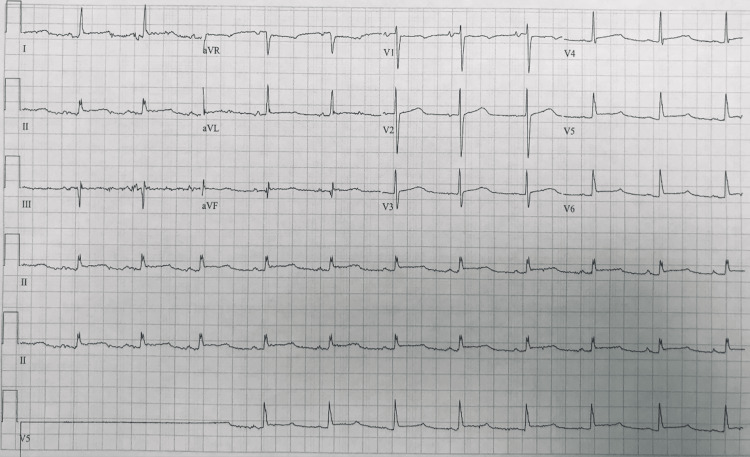
Electrocardiogram showing diffuse ST-segment elevation

**Figure 2:**
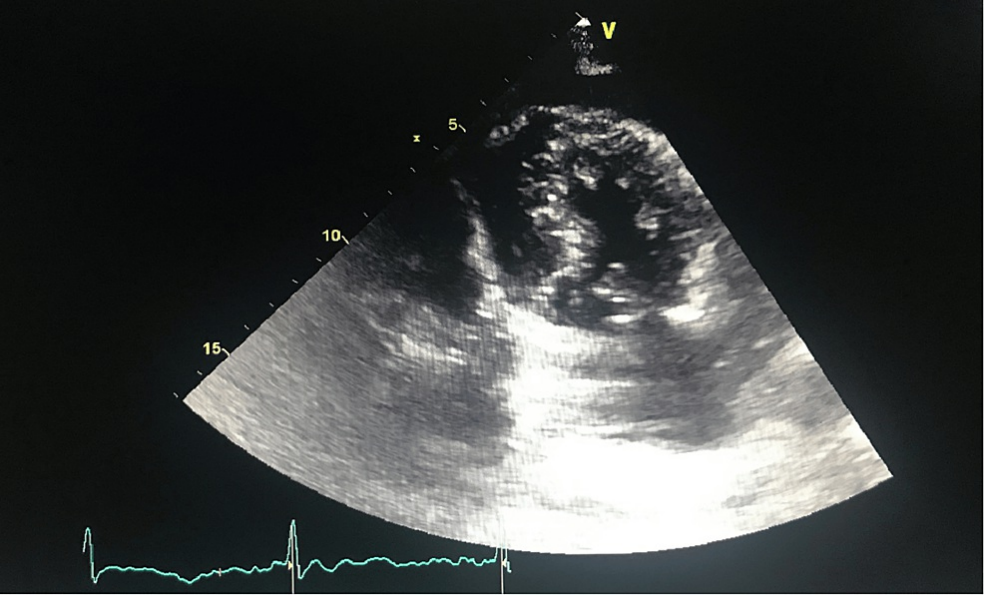
Echocardiogram (apical view) showing evidence of large pericardial effusion with diastolic collapse of the right ventricle

On the eighth day of hospitalization, the patient complained of left-sided burning pleuritic chest pain. An urgent EKG revealed a new diffuse ST segment elevation (Figure 1), and an echocardiogram demonstrated a new large pericardial effusion with the diastolic collapse of the right ventricle (Figure 2). Noticeably, the patient had neck vein distention, but no tachycardia or hypotension. She was taken for an emergent open pericardial window placement to relieve cardiac tamponade. During the procedure, 600 mL of blood was drained. Considering the timeline of the development of a large bloody pericardial effusion following initiation of apixaban, spontaneous hemorrhagic cardiac tamponade attributed to the use of apixaban was diagnosed. The patient was eventually taken off all anticoagulants.

## Discussion

Our case represents a rare complication from the use of DOAC therapy. In 2019, Asad et al. in their similar systematic review found 26 cases of hemorrhagic cardiac tamponade associated with DOACs; rivaroxaban was used in nine of the cases, while only five of the cases were associated with apixaban [4]. In addition, the most common risk factors found in these cases were elevated creatinine, older age, hypertension, male gender, elevated INR, and drug interactions [4], major risk factors also that were present in our patient. In 2022, a systematic review found 41 cases of hemopericardium associated with the use of DOACs with 95% favorable outcomes, but also with cardiac tamponade occurring in 31/41 of them, and 37/41 cases requiring pericardiocentesis [3]. Thus, the current literature supports the rarity of this complication.

Our patient demonstrated chest pain, neck vein distention, and diffuse ST segment elevation on EKG, but she neither developed hypotension nor diminished heart sounds, two of the three common symptoms suggestive of cardiac tamponade known as Beck’s triad [5]. This is in line with current literature showing a sensitivity of near zero for Beck’s triad and a sensitivity of 50% for a single finding of Beck’s triad in cardiac tamponade patients [6].

In considering potential mechanisms involved in this case presentation, impaired hepatic and renal metabolism of apixaban could be the factor. Apixaban is metabolized via the hepatic CYP3A4 enzyme and excreted via renal pathways. The patient was administered amiodarone for atrial fibrillation rate control, and it is a CYP3A4 inhibitor. This could have lowered the metabolism and raised apixaban to supratherapeutic levels [7]. 

CKD can increase bleeding risk, in general due to platelet dysfunction and impaired interaction of von Willebrand factor with GPIIb-IIIa [8]. This patient had a medical history of stage 4 CKD, which already placed him at an increased risk of bleeding. Moreover, renal secretion of apixaban is mediated by p-glycoprotein and amiodarone is an inhibitor of this protein [9]. Amiodarone thus would affect the clearance of apixaban contributing to supratherapeutic levels of the drug and consequently increase the bleeding risk for this patient.

## Conclusions

Hemorrhagic cardiac tamponade is a rare complication of apixaban administration. The potential mechanisms of CYP3A4 and p-glycoprotein metabolic inhibition by amiodarone could explain this association. Our findings suggest that physicians may need to take additional consideration in administering DOACs, especially in patients with renal insufficiency. Although Beck’s triad has been historically described as a syndrome associated with cardiac tamponade, it may not be always present as in our case. Further investigation is warranted to look into other potential variables involved in the occurrence of hemorrhagic cardiac tamponade in CKD patients on apixaban.
